# Off-the-Shelf, Immune-Compatible Human Embryonic Stem Cells Generated Via CRISPR-Mediated Genome Editing

**DOI:** 10.1007/s12015-020-10113-7

**Published:** 2021-01-09

**Authors:** Annie Kim, Kun-Gu Lee, Yeongbeen Kwon, Kang-In Lee, Heung-Mo Yang, Omer Habib, Jihun Kim, Sang-Tae Kim, Sung Joo Kim, Jin-Soo Kim, Dong-Youn Hwang

**Affiliations:** 1 Center for Genome Engineering, Institute for Basic Science, Seoul, Republic of Korea; 2 Department of Chemistry, Seoul National University, Seoul, Republic of Korea; 3 Department of Biomedical Science, Graduate School of CHA University, Seongnam, South Korea; 4 Samsung Advanced Institute for Health Sciences & Technology(SAIHST), Graduate School, Department of Health Sciences & Technology, Sungkyunkwan University, Seoul, South Korea; 5 Transplantation Research Center, Samsung Biomedical Research Institute, Samsung Medical Center, Seoul, Republic of Korea; 6 ToolGen, Inc., Seoul, South Korea; 7 GenNbio Inc., Seoul, South Korea; 8 Department of Medicine, Sungkyunkwan University School of Medicine, Suwon, South Korea; 9 Department of Chemistry, Hanyang University, Seoul, Republic of Korea; 10 BotBot Inc., Seoul, South Korea; 11 Department of Life Sciences, The Catholic University of Korea, Bucheon-si, Gyeonggi-do South Korea

**Keywords:** Human embryonic stem cells, HLA-editing, CRISPR/Cas9, HLA homozygous-like hESCs, Immune-compatible hESC banking

## Abstract

Human embryonic stem cells (hESCs) hold promise in regenerative medicine but allogeneic immune rejections caused by highly polymorphic human leukocyte antigens (HLAs) remain a barrier to their clinical applications. Here, we used a CRISPR/Cas9-mediated HLA-editing strategy to generate a variety of HLA homozygous-like hESC lines from pre-established hESC lines. We edited four pre-established HLA-heterozygous hESC lines and created a mini library of 14 HLA-edited hESC lines in which single HLA-A and HLA-B alleles and both HLA-DR alleles are disrupted. The HLA-edited hESC derivatives elicited both low T cell- and low NK cell-mediated immune responses. Our library would cover about 40% of the Asian-Pacific population. We estimate that HLA-editing of only 19 pre-established hESC lines would give rise to 46 different hESC lines to cover 90% of the Asian-Pacific population. This study offers an opportunity to generate an off-the-shelf HLA-compatible hESC bank, available for immune-compatible cell transplantation, without embryo destruction.

**Supplementary Information:**

The online version contains supplementary material available at 10.1007/s12015-020-10113-7.

**Figure:**
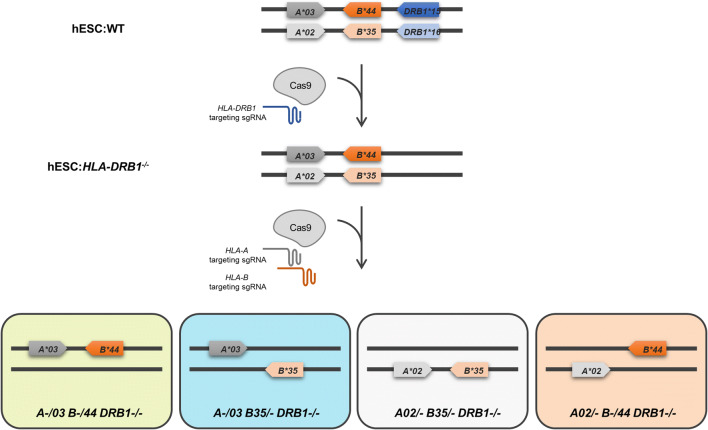
Graphical Abstract

## Introduction

Human embryonic stem cells (hESCs) hold great promise for cell replacement therapy. HESCs derived from the inner cell mass (ICM) of pre-implantation blastocyst-stage embryos retain pluripotency in vitro. The advent of hESCs opened a new era in regenerative medicine because, under appropriate conditions, hESCs can be coaxed into any cell type of the three lineages, ectoderm, mesoderm, and endoderm. These pluripotent characteristics make hESCs a valuable cell resource in regenerative medicine.

Despite their tremendous potential, immune rejection of allogeneic hESC-derived cells remains the major obstacle for the clinical usage of these cells [1]. Cell surface expression of human leukocyte antigens (HLAs) are the major immunologic barrier in cell transplantation [1]. A number of studies have reported better survival of patients who received solid organ transplants derived from HLA-A, -B, and -DR matched donors [2]. However, it is difficult to find donor organs or cells that are HLA-compatible at these three loci for transplantation [1, 3–5]. Although immunosuppressive drugs can be used to prevent rejection, prolonged immunosuppression increases the risk of serious side effects such as cancer and infection [6].

Autologous, induced pluripotent stem cells (iPSCs) could, in principle, bypass allogeneic immune rejection. However, derivation of patient-specific iPSCs would not be a practical option, especially in cases of urgent need, due to the high cost and time-consuming nature of iPSC generation. More importantly, genomic and epigenetic abnormalities occurring during the reprogramming process would be critical issues that must be resolved before iPSCs could be used in the clinic [7–12].

A practical solution to the immune-rejection/compatibility issue would be to create a bank of hESC lines that encompass a broad spectrum of HLA types. Some HLA haplotypes are common in certain populations and establishment of HLA-homozygous hESC lines with common haplotypes would decrease the number of hESC lines needed for banking. One study estimated that 55 parthenogenetic HLA-homozygous hESC lines derived from randomly donated embryos could provide a full match of HLA-A, -B, and -DR for 80% of the Japanese population [13]. However, because of the limited availability of donated embryos and associated ethical issues, it would be extremely challenging to generate enough HLA-homozygous hESC lines, either parthenogenetic or normal, to match a significant proportion of the population.

An alternative solution for avoiding immune rejection of hESC derivatives is to genetically manipulate HLA genes. By using zinc finger nucleases, Torikai and colleagues selectively eliminated HLA-A expression in T-cells and hESCs [14] and Riolobos and colleagues disrupted beta-2 microglobulin (B2M), which encodes a component of HLA class I molecules, and thus prevented the surface presentation of all HLA class I molecules [15]. More recently, cytotoxic T lymphocyte-associated protein 4 (CTLA4)-immunoglobulin and programmed cell death ligand 1 (PD-L1) knock-in hESCs were generated and were shown to suppress the host immune response [16].

Based on previous studies, attempts have been made to generate universal donor human pluripotent stem cells (PSCs) that evade immune surveillance by knocking out B2M and/or CIITA, a transcription factor of HLA class II genes, to ablate surface presentation of HLA class I and/or II [17–22]. To allow the universal donor cells to evade host immune surveillance, several immune regulatory genes, such as those encoding HLA-E, -G, CD47, and PD-L1, were introduced [17–19]. Very recently, using iPSCs, Xu et al. disrupted two major classical HLA class I molecules (i.e., by biallelic knockout of the *HLA-A* and *B* genes and monoallelic knockout of the *HLA-C* gene) and all HLA class II molecules (by knockout of the *CTIIA* gene) [22].

Although these approaches certainly enhanced the survival of the genome-engineered cells in vitro and in humanized mice, it remains to be scrutinized whether extensive disruption of HLA class I and II genes by knocking out *B2M* and *CIITA*, respectively, affects proper antigen presentation and the function of a variety of interacting proteins, which would result in unwanted side effects in a human trial [23, 24]. For example, knocking out B2M may negatively affect the cells, because B2M has been shown to interact with many proteins, such as cluster of differentiation 1, homeostatic iron regulator, leukocyte immunoglobulin-like receptor, Fc fragment of IgG receptor and transporter, and major histocompatibility complex class I-related gene protein. In addition, complete removal of some HLAs and full immune suppression may increase the risk of tumorigenesis and pathogenic infection of the transplanted cells [25].

In our study, we used the similar HLA-matching strategy to that used in traditional organ transplantation (i.e., matching of HLA-A, B, and DRB1) and introduced only modest genetic engineering of HLA genes: we generated mono-allelic ablations of the *HLA-A* and *HLA-B* genes and bi-allelic ablations of the *HLA-DRB1* gene using CRISPR/Cas9 nucleases [26]. The resulting HLA-A, -B hemizygous (+/−) and HLA-DRB1 homozygous knockout (−/−) hESCs suppressed natural killer (NK) and T cell recognition, offering a solution to allogeneic immune rejections. We also showed that a small library of HLA-A, -B hemizygous hESCs derived from pre-established hESC lines could cover a significant proportion of several human populations.

Therefore, this study provides an efficient platform for establishing an hESC bank for “off-the-shelf” immune-compatible cell therapy.

## Materials and Methods

### HLA Typing of hESC Lines

HLA haplotype information for the CHA6, CHA15, and H9 lines was obtained from the literature [27, 28]. For the SNU31 line, HLA typing analysis was performed by PCR-sequencing based typing (PCR-SBT) (DowGene DNA Testing Co., Seoul, Korea). The types of *HLA-A*, *-B*, and *-DRB1* genes were confirmed by sequencing of PCR amplicons of each gene.

### Cell Culture

Human ESCs were cultured in TeSR™-E8™ medium (STEMCELL Technologies, Vancouver, BC, Canada) on culture dishes coated with matrigel (Thermo Fisher Scientific, Waltham, MA, USA). Passaging of the cells was performed every 5 days by dissociating hESC colonies with EDTA (Thermo Fisher Scientific) for 4 min at 37 °C followed by replating on freshly coated dishes.

### Karyotyping

Karyotyping analysis of hESCs was performed at CANCERROP Inc. (Seoul, Korea).

### Cas9 Protein Purification and In Vitro sgRNA Transcription

Recombinant Cas9 protein was purchased from ToolGen Inc. (Seoul, Korea). Single guide RNAs (sgRNAs) were synthesized by in vitro transcription using T7 RNA polymerase (New England BioLabs, Ipswich, MA USA) as previously described (Kim et al., 2017). In brief, sgRNA templates were incubated with T7 RNA polymerase in reaction buffer (40 mM Tris-HCl, 20 mM MgCl_2_, 1 mM DTT, 2 mM spermidine, at pH 7.9) (New England Biolabs), containing 4 mM NTPs (Jena Bioscience, Jena, Germany) and RNase inhibitor (New England Biolabs) for 16 h at 37 °C, followed by incubation with DNase I (New England Biolabs) for 30 min at 37 °C. Synthesized sgRNAs were purified with a PCR purification kit (Macrogen, Seoul, Korea). The sequences of oligonucleotides used for sgRNA template synthesis are shown in Table S1.

### Cas9-Ribonucleoprotein Delivery

To edit HLA genes in hESCs, cells were transfected with a Cas9-ribonucleoprotein (RNP) complex using a 4D-Nucleofector (Lonza, Basel, Switzerland). The RNP complex was made by mixing 10 μg of Cas9 protein in storage buffer (20 mM HEPES pH 7.5, 150 mM KCl, 1 mM DTT, and 10% glycerol) with 7.5 μg of in vitro transcribed sgRNAs and then incubating the mixture for 15 min at room temperature. We used 1 X 10^5^ hESCs in 20 μl Primary P3 buffer and performed electroporation using the nucleofector program CB-150. After transfection, the cells were plated on Geltrex (Thermo Fisher Scientific)-coated 24-well plates and cultured in Essential 8™ Flex Medium containing RevitaCell Supplement (Thermo Fisher Scientific).

### Targeted Deep Sequencing

For target site amplification, 100 ng of genomic DNA was amplified with KAPA HiFi HotStart PCR Polymerase (Roche, Basel, Switzerland). For deep sequencing library generation, amplicons were amplified a second time using TruSeq HT Dual Index Primers (Illumina, San Diego, CA, USA). Paired-end sequencing was performed using the Illumina Miniseq System. Indel frequencies were calculated at https://github.com/ibs-cge/maund.

### Whole Genome Sequencing

Genomic DNAs from H9-hESC and HLA-edited hESC lines were used for whole genome sequencing. Sequencing was performed with an Illumina HiSeq X10 Sequencer at a sequencing depth of 30X to 40X (Macrogen). Total variants were called using the Isaac Variant Caller and annotated variants were filtered out by SnpEff. Potential off-target site candidates were obtained using Cas-OFFinder (http://www.rgenome.net/). We searched for potential off-target sites by allowing mismatches up to 7-bp in length or 5-bp in length with 2-bp DNA or RNA bulges. Then, we compared the candidates to the unique single nucleotide polymorphisms in the HLA-edited hESC clones.

### Expansion and Staining of Activated NK Cells

Whole blood samples were collected from healthy donors (IRB No. SMC 2018-12-021). Peripheral blood mononuclear cells (PBMCs) were isolated from the whole blood by Ficoll density gradient centrifugation and were screened to determine the HLA-type. The PBMCs were then cultured using an NK cell expansion medium system (KBM NK Kit) (Kohjin Bio, Sakado, Japan) for a total of 10–14 days according to the manufacturer’s protocol. Ultimately, highly pure NK cells were isolated using CD56 MicroBeads (Miltenyi Biotec Inc., Auburn, CA, USA) with a VarioMACS system (Miltenyi Biotec Inc.). Cultured NK cells were harvested and analyzed by flow cytometry to determine their purity, and CD3^−^CD56^+^ NK cells were determined to be 90–95% pure.

The following monoclonal antibodies were used to stain cultured NK cells: anti-CD45-BUV395 (1:200, Clone HI30, BD Biosciences), anti-CD3-APC-Cy7 (1:100, Clone SP34–2, BD Biosciences), anti-CD56-PE (1:100, Clone CMSSB, Invitrogen**)**, anti-CD16-BV421 (1:100, Clone 3G8, BD Biosciences), anti-CD19-PE-CF594 (1:200, Clone HIB19, BD Biosciences), anti-CD14-PE-CF594 (1:200, Clone MQP9, BD Biosciences), anti-NKG2D-APC (1:100, Clone 1D11, Invitrogen), anti-NKp46-BV510 (1:100, Clone 9E2, Biolegend), anti-CD94-PerCP-Cy5.5 (1:100, Clone DX22, Biolegend), and anti-CD69-PE-Cy7 (1:100, Clone FN50, Invitrogen).

### In Vitro Fluorometric Cytotoxicity Assays to Measure NK Cell Effects, Including a CD107a Degranulation Assay

The effects of NK cells on HLA-edited target cells and K562 cells (ATCC) were assessed by flow cytometry. Target cells were stained with CellTraceRed (Thermo Fisher Scientific). Activated NK cells were labeled with CellTraceViolet (Thermo Fisher Scientific) for 15 min at room temperature, and were co-cultured with target cells at various ratios for 4 h in the presence of anti-CD107a-PE (1:50, Clone H4A3, BD Biosciences), BD GolgiStopTM (BD Biosciences), and BD GolgiPlugTM (BD Biosciences) in a 37 °C 5% CO_2_ incubator. After incubation, the cells were washed with 1X phosphate-buffered saline (PBS). To measure the number of apoptotic target cells, the washed cells were stained with 7-Amino-Actinomycin (7-AAD, BD Bioscience) following the manufacturer’s instructions and analyzed with an LSR Fortessa cell analyzer (BD Biosciences). Next, to assess the NK cell cytotoxicity, the harvested cells were stained with Live/Dead reagents (1:200, Invitrogen) and antibodies recognizing surface molecules. The following monoclonal antibodies were used to stain NK cells: anti-CD45-BUV395 (1:200, Clone HI30, BD Biosciences), anti-CD3-V500 (1:100, Clone SP34-2, BD Biosciences), and anti-CD56-APC-eFluor 780 (1:100, Clone CMSSB, Invitrogen). Then, the cells were permeabilized with a BD CytoFix/CytoPerm Kit (BD Biosciences) and intracellular cytokines were stained with anti-IFN-γ-BV605 (1:100, Clone B27, BD Biosciences) and anti-TNF- alpha -PE-Cy7 (1:100, Clone Mab11, BD Biosciences). Stained cells were analyzed with an LSR Fortessa cell analyzer (BD Biosciences) and the results were analyzed using FlowJo software (BD Biosciences).

### Expansion of Activated T Cells

To expand activated T cells, PBMCs were added to flasks pre-coated with anti-CD3 (1:500, Clone OKT-3, Invitrogen) and anti-CD28 (1:500, Clone CD28.2, Invitrogen) monoclonal antibodies and then incubated in serum-free KBM551 medium (Kohjin Bio) containing 200 IU/ml human recombinant interleukin-2 (rhIL-2) (R&D Systems) at 37 °C in a 5% CO_2_ atmosphere. Four days later, the cells were transferred into a culture bag (Kohjin Bio) and were cultured for a total of 10–14 days. Cultured T cells were harvested and analyzed by flow cytometry. The following monoclonal antibodies were used to stain cultured T cells: anti-CD45-BUV395 (1:200, Clone HI30, BD Biosciences), anti-CD3-PE-Cy7 (1:100, Clone SP34-2, BD Biosciences), anti-CD56-PE (1:100, Clone CMSSB, Invitrogen), anti-CD19-PE-CF594 (1:200, Clone HIB19, BD Biosciences), anti-CD14-PE-CF594 (1:200, Clone MQP9, BD Biosciences), anti-CD4-BV605 (1:100, Clone RPA-T4, BD Biosciences), anti-CD8-APC-R700 (1:100, Clone SK1, BD Biosciences), anti-CCR7-PerCP-Cy5.5 (1:100, Clone G043H7, Biolegend), and anti-CD45RA-APC-H7 (1:100, Clone HI100, BD Biosciences).

### In Vitro T Cell Proliferation and Cytokine Production Assay

The HLA-edited target cells were pretreated with 100 ng/ml interferon-γ (IFN-γ) (Peprotech, Rocky Hill, NJ, USA) for 48 h, followed by treatment with 10 μg/ml mitomycin C (Tocris, Abingdon, United Kingdom) for 1.5–2 h, after which they were irradiated (40 Gy) to stop proliferation. Because they have diverse HLA antigens, third party PBMCs were pooled from ten healthy donors for use as a positive control. Target cells were labeled with CellTrace Red (Thermo Fisher Scientific) to distinguish them from responder T cells that were labeled with CellTrace Violet (Thermo Fisher Scientific). After labeling, target cells were co-cultured with responder T cells at a 1:1 ratio in the presence of anti-CD107a-PE and 20 U/ml interleukin-2 (IL-2). Plates (96-well) containing 1 × 10^5^ cells/well were maintained in a 37 °C CO_2_ incubator for 6 days. On the day of harvest, anti-CD3 (1:500, Clone OKT-3, Invitrogen) and anti-CD28 (1:500, Clone CD28.2, Invitrogen) monoclonal antibody were added to the plates, followed by treatment with GolgiPlug™ and GolgiStop™ (BD Biosciences) 1 h later. After 5-h treatment with GolgiPlug™ and GolgiStop™, T cell proliferation was analyzed by assessing the percentage of CTV^low^ dividing cells to obtain the mitotic index (Tanaka et al., 2004). Incubated cells were stained with the following monoclonal antibodies: anti-CD45-BUV395 (1:200, Clone HI30, BD biosciences), anti-CD3-V500 (1:100, Clone SP34–2, BD Biosciences), anti-CD4-PerCP-Cy5.5 (1:100, Clone RPA-T4, BD biosciences), and anti-CD8-APC-R700 (1:100, Clone SK1, BD Biosciences). Then, surface stained cells were permeabilized with BD CytoFix/CytoPerm Kit (BD Biosciences), and intracellular cytokines were stained with anti-IFN-γ-BV605 and anti-TNF-alpha-PE-Cy7. Finally, stained cells were analyzed with an LSR Fortessa instrument and the result were analyzed using FlowJo software.

### Coverage Calculation

To estimate the population coverage, we used the allele frequency to calculate the HLA-AB haplotype combinations. Allele frequency information was acquired from a previous report [29]. Based on this information, we made a computer program to predict haplotype frequency and the accumulative coverage. The source code of the program and the haplotype frequency calculation are available at https://github.com/ibs-cge/gene_coverages.

### Endothelial Cell Differentiation

Human ESCs were seeded on 6-well plates (Thermo Fisher Scientific) and cultured in TeSR™-E8™ medium (STEMCELL Technologies) containing 10 mM ROCK inhibitor, Y-27632 (Tocris, Abingdon, United Kingdom), for 1 day. The following day, the medium was changed to N2B27 medium containing 46.9% (*v*/v) Dulbecco’s Modified Eagle Medium/F12 (Gibco, Rockville, MD, USA), 50% (v/v) Neurobasal medium (Gibco), 0.97% N2 (Gibco), 1.94% B27 (Gibco), 0.97% β-mercaptoethanol (Gibco), 8 μM CHIR99021 (Cayman Chemical), and 25 ng/ml bone morphogenetic protein-4 (Peprotech), after which the cells were incubated for 3 days. The medium was then replaced with StemPro-34 SFM (Thermo Fisher Scientific) supplemented with 100 ng/ml vascular endothelial growth factor (Peprotech), 50 ng/ml basic fibroblast growth factor (CHA Meditech, Seongnam, Korea), and 2 μM forskolin (Abcam, Cambridge, United Kingdom), and cells were incubated for 2 days. The cell population was then enriched for CD144^+^ cells using MACS cell separation (Miltenyi Biotec). The CD144^+^ cells were plated and maintained in matrigel-coated plates containing endothelial cell growth medium (EGM™-2 BulletKit™) (Lonza, Basel, Switzerland) for further differentiation for at least 7 days. Endothelial cells were analyzed by flow cytometry using anti-CD31 antibodies (1:25, Clone WM-59,eBioscience) and by immunocytochemistry with anti-CD31 antibodies (1:200, Clone HC1/6, Millipore) and anti-Von Willebrand Factor antibodies (1:200, Abcam).

### Immunocytochemistry

Cells were fixed in 4% paraformaldehyde in PBS for 10 min and then permeabilized with 0.1% Triton X-100 (Sigma-Aldrich, St. Louis, Missouri, USA) for 5 min. Cells were blocked with 5% normal goat serum (Vector Laboratories, Burlingame, CA, USA), 1% bovine serum albumin (BSA) (BOVOGEN, East Keilor, Australia), and 0.1% Tween-20 (Sigma-Aldrich) in PBS for 1 h at room temperature. The cells were then incubated with the primary antibodies for 3 h at 37 °C or overnight at 4 °C. Secondary antibodies used were Alexa Fluor 488 (1:200, Invitrogen), Alexa Fluor 555 (1:200, Invitrogen), and Alexa Fluor 594 (1:200, Invitrogen). The samples were then treated with 4′,6-diamidino-2-phenylindole (Sigma-Aldrich) for 10 min. Images were visualized with a Zeiss LSM880 confocal microscope (Zeiss).

### Quantitative Reverse Transcriptase-Polymerase Chain Reaction (RT-qPCR)

Total RNA was extracted using a NucleoSpin RNA II Kit (MACHEREY-NAGEL, Düren, Germany) according to the manufacturer’s instructions. Complementary DNA (cDNA) was synthesized from 1 μg of total RNA using a ReverTra Ace qPCR RT Kit (Toyobo, Osaka, Japan) according to the manufacturer’s instructions. qRT-PCR analysis was performed using Power SYBR Green PCR Master Mix (Applied Biosystems, Forster City, California, USA) and a StepOnePlus Real-Time PCR System (Applied Biosystems) under the following conditions: 40 cycles of DNA denaturation at 95 °C for 5 s, DNA annealing with each primer pair at 55–63 °C for 30 s, and polymerization at 72 °C for 30 s. The primers used in this study are listed in Table S2.

### HLA-A Immunocytochemistry by Flow Cytometry

Cells were detached from plates by treatment with Accutase (Thermo Fisher Scientific) for 5 min at 37 °C. Single cell suspensions were obtained by passing the suspension through a 45-μm cell strainer (BD Biosciences). PBS containing 1% BSA was used as washing and staining buffer. Briefly, dissociated single cells were washed and then stained with antibodies at 4 °C for 30–60 min. Samples were analyzed on a CytoFlex flow cytometer (Beckman Coulter, Brea, California, USA). The data were plotted using FlowJo software. The antibodies used were as follows: anti-HLA class I A2 A28 antibody (ab31568, clone 8.L.125), anti-HLA class I A3 antibody (ab31572, clone 4i153), isotype controls mouse IgG2a kappa (ab170191), and mouse IgM lambda (ab18396) (all Abcam). The amount of the antibodies used in this experiment was in accordance with the manufacturer’s guideline.

### Quantification and Statistical Analysis

Statistical details are described in the figure legends. All statistical tests were performed using GraphPad Prism 7 (GraphPad Software). A *P* value less than 0.05 was considered significant.

## Results

### Generation of HLA-Engineered hESC Lines

For overcoming immunological barriers in cell and organ transplantation, the most important HLA molecules to be matched are HLA-A and HLA-B of class I and HLA-DR of class II [1]. In an effort to establish a cell banking platform, we sought to generate and test the efficacy of HLA-A and B hemizygous (homozygous-like) hESC lines, which allow immune-compatible cell therapy for many people. To this end, we used CRISPR/Cas9 genome-editing technology to establish hESC lines with a biallelic knockout of the *HLA-DRB1* gene and monoallelic knockout of the *HLA-A* and *-B* genes. We chose four hESC lines, H9, CHA15, CHA6, and SNU31, with different HLA haplotypes, to obtain hESC lines with diverse HLA-A and -B hemizygosity: After editing the *HLA* genes, we could obtain 14 non-overlapping HLA-A and -B hemizygous library lines as shown in Fig. S1a. In our CRISPR/Cas9-mediated *HLA* gene editing, we designed subtype-specific sgRNAs using the IPD and IPD-IMGT/HLA Databases [30] (Fig. S1b).

**Fig. 1:**
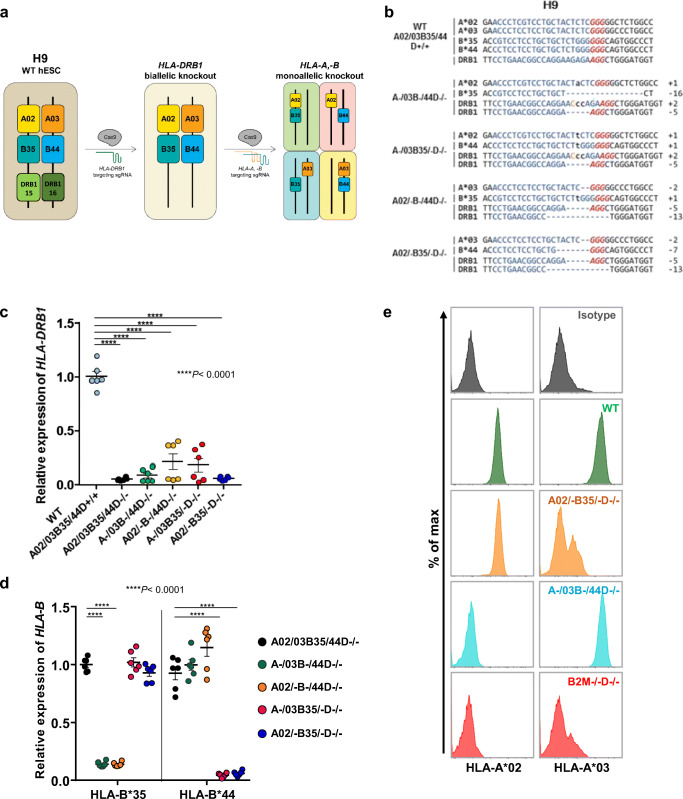
Generation an HLA-A, B hemizygous hESC library and validation of knockout cells. (**a**) Schematic diagram of the HLA-DRB1 (null), HLA-A (hemizygous), and HLA-B (hemizygous) knockout strategy in H9 hESCs. The *HLA-DRB1* gene was biallelically targeted by transient delivery of preassembled single-chain guide RNA (sgRNA)/Cas9 protein ribonucleoproteins (RNPs), followed by the monoallelic knockout of the *HLA-A* and *HLA-B* genes. (**b**) Target sites and indel patterns of the isolated mutant clones derived from H9 hESCs. The protospacer adjacent motif and the sgRNA target sequence are shown in red and blue, respectively. Deletions and insertions of nucleotides are indicated as dashes and lowercase letters, respectively. Nucleotide changes are shown in orange upper case letters. (**c**) Relative HLA-DRB1 mRNA levels in H9_WT, HLA-DRB1 null, and HLA-AB hemizygous hESC clones were compared. The level of HLA-DRB1 mRNA in H9_WT cells was arbitrarily set to 1. Ordinary one-way ANOVA was performed, followed by Tukey’s multiple comparison test. ****P < 0.0001. (**d**) Relative HLA-B mRNA levels in HLA-DRB1 null and HLA-AB hemizygous hESC clones were compared. Two-way ANOVA followed by Tukey’s multiple comparison test was performed. Cells were treated with 100 ng/ml IFN-γ for 48 h before the experiment and HLA-DRB1 and HLA-B expression was measured by RT-qPCR. The level of HLA-B mRNA in the H9 A02/03B35/44D−/− line was arbitrarily set to 1. Means ± SEM were from six independent experiments. ****p < 0.0001. (**e**) HLA-A*02 and HLA-A*03 expression levels in the H9_WT, A02/−B35/−D−/−, A−/03B−/44D−/−, and B2M−/−D−/− hESC clones were measured by flow cytometry. Cells were treated with 100 ng/ml IFN-γ for 48 h before the experiment. See Fig. S2D for the B2M−/− D−/− indel pattern

Because HLA class II antigens are expressed primarily on antigen presenting cells such as B-lymphocytes, macrophages, and dendritic cells, and, unlike class I antigens, their loss does not trigger NK cell and T cell-mediated cell lysis, we first disrupted both *HLA-DRB1* alleles (Fig. 1a). Next, using the HLA-DRB1 biallelic knockout clones, we generated various monoallelic HLA-A, B knockout hESC lines by co-transfecting haplotype-specific HLA-A and HLA-B sgRNAs in various combinations along with the Cas9 protein (Fig. 1a). After each round of genome-editing, we performed targeted deep sequencing to analyze indel patterns and selected frame-shifted knockout clones for further experiments (Fig. 1b, S2).

The HLA-edited clones were validated by examining the expression of HLA molecules (HLA-A, -B, and -DRB1). First, the clones were treated with IFN-γ to boost HLA gene expression [31, 32], after which they were subjected to quantitative RT-PCR using primer pairs recognizing both alleles of the *HLA-DRB1* or *-B* genes (Fig. 1c–d and Table S1). Because of a lack of antibodies recognizing the HLA-DRB1 and HLA-B protein subtypes affected in our HLA-edited clones, we performed quantitative RT-PCR analysis to assess *HLA-DRB1* or *-B* gene expression: It has been well documented that the introduction of a premature stop codon results in a dramatic reduction in the mRNA level via the nonsense-mediated mRNA decay pathway [33, 34]. Unlike wild-type (WT) H9 hESCs, all of the HLA-edited clones showed almost no HLA-DRB1 expression (Fig. 1c). With allele-specific PCR primers, the HLA-B mRNA level of each clone was examined. As expected, only the alleles with an indel mutation did not express the *HLA-B* gene (Fig. 1d).

To measure the expression of each *HLA-A* allele, we performed flow cytometric analysis using allele-specific HLA-A antibodies. We observed that the targeted alleles showed almost undetectable HLA-A expression, similar to the lack of expression seen in B2M−/−D−/− H9 hESCs (Fig. 1e).

**Fig. 2:**
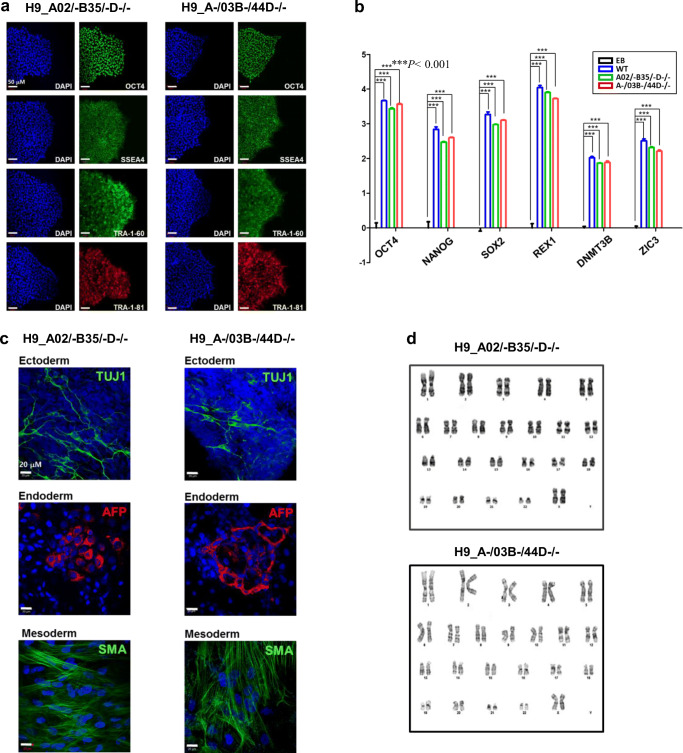
Characterization of HLA-edited hESC clones. (**a**) The HLA-edited hESC clones used for T cell response analysis, A02/−B35/−D−/− and A−/03B−/44D−/−, were positive for the pluripotent cell markers OCT4, SSEA4, TRA-1-60, and TRA-1-81. Scale bar: 50 μm. (**b**) Quantitative real-time RT-PCR was performed to detect the expression of the representative pluripotency markers, OCT4, NANOG, SOX2, REX1, DNMT3B, and ZIC3. Data are represented as means ± SEMs (*n* = 3). ***p < 0.001, two way ANOVA, followed by Tukey’s multiple comparison test. (**c**) HLA-edited hESC clones were spontaneously differentiated into cell derivatives of the three germ layers, and the expression levels of representative markers of the ectoderm (TUJ1), mesoderm (SMA), and endoderm (AFP) lineages were examined. (**d**) Karyotyping analysis was performed to detect gross chromosomal abnormalities

The HLA-edited hESC lines retained typical characteristics of pluripotent stem cells: They expressed various pluripotent cell markers (Fig. 2a, b) and retained the ability to differentiate into three lineages (Fig. 2c). Furthermore, no gross chromosomal aberrations were detected (Fig. 2d). To examine the integrity of the genome in more detail, we performed whole genome sequencing and found no genomic abnormalities (Fig. S3).

Together, these results show that we established a method for generating HLA-edited hESC lines for immune-compatible cell banking by mutating the *HLA-A* (monoallelic knockout), *HLA-B* (monoallelic knockout), and *HLA-DRB1* (biallelic knockout) genes.

### Activated NK Cell Response to the HLA-Edited Cells

**Fig. 3:**
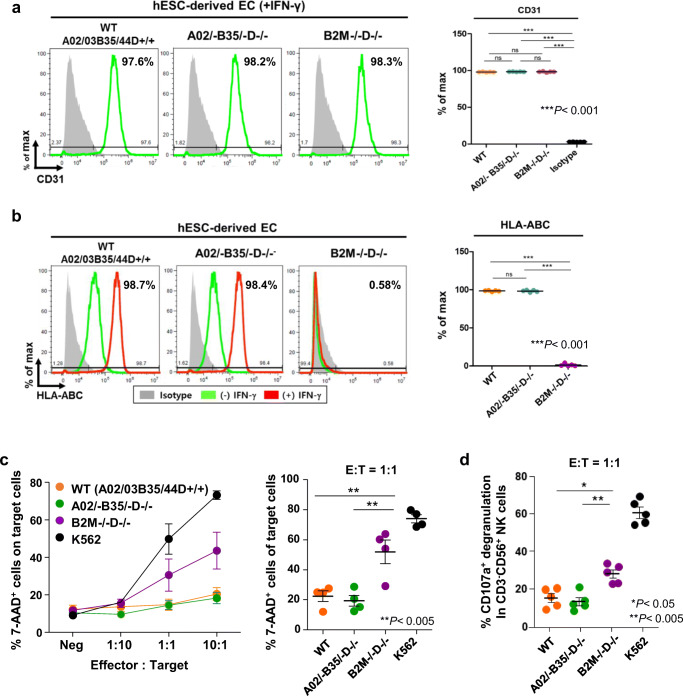
Genome-edited HLA-A, B hemizygous and -DRB1 null cells evade NK cell activity in vitro*.* (**a**) Cell-surface CD31 expression levels on IFN-γ-treated ECs differentiated from H9_WT, A02/−B35/−D−/−, and B2M−/−D−/− hESC lines. Data are represented as means ± SEMs (*n* = 5). ***p < 0.001, one-way ANOVA, followed by Tukey’s test. (**b**) Flow cytometric analysis of HLA-ABC expression on ECs derived from H9_WT, A02/−B35/−D−/−, and B2M−/−D−/− hESC lines. The expression of HLA-ABC was examined with or without IFN-γ treatment. Data are represented as means ± SEMs (n = 5). ***p < 0.001, one-way ANOVA, followed by Tukey’s test. (**c**) 7-AAD assay to measure the percentage of apoptotic target cells in EC populations differentiated from H9_WT, A02/−B35/−D−/−, B2M−/−D−/− hESC lines after incubation with HLA null-reactive NK cells (derived from 3 donors) at various effector: target (E:T) ratios (*left panel*). The percentage of 7-AAD^+^ apoptotic target cells among different target EC populations when treated at an E:T ratio of 1:1 is shown (*right panel*). (**d**) CD107a expression on CD3^−^CD56^+^ NK cells was measured by flow cytometry after co-culture with various types of target cells at an E:T ratio of 1:1. Data are represented as means ± SEMs. *p < 0.05, and ** *p* < 0.005, followed by Mann-Whitney nonparametric test

To evaluate the immunogenicity of the HLA-edited cells, we differentiated WT and HLA-edited hESCs into endothelial cells (ECs) and observed that about 98% of the cells expressed a typical EC marker, CD31^+^ (Fig. 3a). Immunostaining also confirmed that these hESC-derived ECs expressed typical markers, CD31 and Von Willebrand factor (Fig. S4). The resulting WT and HLA-edited ECs presented HLA-ABC antigens on the cell surface, the level of which was further increased by IFN-γ stimulation (treatment with 100 ng/ml IFN-γ for 48 h) (Fig. 3b). In contrast, no cell surface presentation of HLA-ABC antigens was detected on B2M-knockout (B2M−/−D−/−) ECs with or without IFN-γ stimulation (Fig. 3b).

We first assessed the in vitro cytotoxicity of expanded NK cells by co-culturing them with CD31^+^ ECs derived from H9-WT (A02/03B35/44DRB1+/+), HLA-edited (A02/−B35/−D−/−), and B2M knockout (B2M−/−DRB1−/−) hESCs, as well as with K562, a human leukemia cell line that lacks the HLA complex.

For this experiment, we first established an optimal expansion protocol for activated NK cells. After ex vivo expansion for 2 weeks, we observed a remarkable increase in the CD3^−^CD56^+^ NK cell population (from 7.85% to 73.00%) (Fig. S5a). The expression of several NK cell activating receptors including CD16, NKG2D, CD94, and CD69 were upregulated after the 2 week expansion compared to before expansion (Fig. S5b).

We assessed the cytotoxicity of effector NK cells using flow cytometry, and apoptotic cells were detected as the 7-Aminoactinomycin D^+^ (7-AAD^+^) cells within the CellTrace Red^+^ (CTR^+^) gate: CTR-labeled target cells (ECs) were discriminated from CellTrace Violet (CTV)-labeled NK cells. NK cell-mediated cytotoxicity for HLA-AB hemizygous ECs (e.g., A02/–B35/−D−/−) and H9_WT hESC-derived ECs was comparable (Fig. 3c). In contrast, class I HLA-lacking B2M knockout (B2M−/−DRB1−/−) ECs and HLA-devoid K562 tumor cells were significantly vulnerable to NK cell cytotoxicity (Fig. 3c). Furthermore, the NK cell cytotoxicity to these two types of cells increased as the effector: target (E:T) cell ratio became larger. These results show that HLA-AB hemizygous (homozygous-like) ECs do not show increased susceptibility to cell death caused by NK cells, compared with H9_WT hESC-derived ECs (Fig. 3c).

Next, we examined the cytokine production and degranulation activity of NK cells in response to HLA-edited ECs. CD107a expression on NK cells is known to be a prominent marker of NK cell degranulation, and positively correlated with cytokine production and NK cell-mediated killing of target cells [35–37]. Degranulation of NK cells was significantly higher for B2M−/−DRB1−/− target cells than for WT and A02/−B35/−D−/− ECs (Fig. 3d). Furthermore, we measured cytokine secretion by NK cells in response to HLA-edited target cells (Fig. S6). The secretion of IFN-γ and tumor necrosis factor-α (TNF-α) was not significantly different in the presence of WT versus HLA-AB hemizygous (A02/−B35/−D−/−) cells at an E:T cell ratio of 1:1. Altogether, these results show that HLA-AB hemizygous cells evade NK cell cytotoxicity as efficiently as WT cells.

### T Cell Response to the HLA-Edited Cells

To determine whether activated T cells selectively react to HLA-edited target cells, we established mixed lymphocyte reactions (MLRs) in the presence of a low dose of IL-2. Two types of HLA-edited target cells, A02/−B35/−D−/− and A−/03B−/44D−/− ECs, retained matched and unmatched HLA types, respectively, with the responder PBMCs. Information about the HLA-A, B, and DRB1 type of the responder PBMCs and 3rd party pooled control PBMCs is shown in Fig. S7a. For this experiment, we first differentiated the two HLA-edited hESC lines (A02/−B35/−D−/− and A−/03B−/44D−/−) into target CD31^+^ ECs (Fig. S7b), and then pretreated the cells with IFN-γ to boost the expression of HLA molecules (Fig. S7c). The responder cells were generated by expansion and activation of T cells from PBMCs using anti-CD3, anti-CD28, and IL-2 for 2 weeks: After T cell expansion, CD3^+^ T cells were enriched and only a few CD3^−^CD56^+^ NK cells were present (Fig. S7d, *left column*). A large portion of the expanded CD3^+^ T cells were CD8^+^ cytotoxic T cells (60.9%, 44.1%, and 62.4% from three experiments) (Fig. S7d, *middle column*). Furthermore, the expanded T cell population contained many CCR7^−^CD45RA^−^ effector-memory (T_EM_) and CCR7^−^CD45RA^+^ terminally differentiated memory (T_EMRA_) T cells (Fig. S7d, *right column*). CD45RA^+^ effector T_EMRA_ cells are known to have less proliferative potential but produce cytokines and exert competent effector functions [38].

**Fig. 4:**
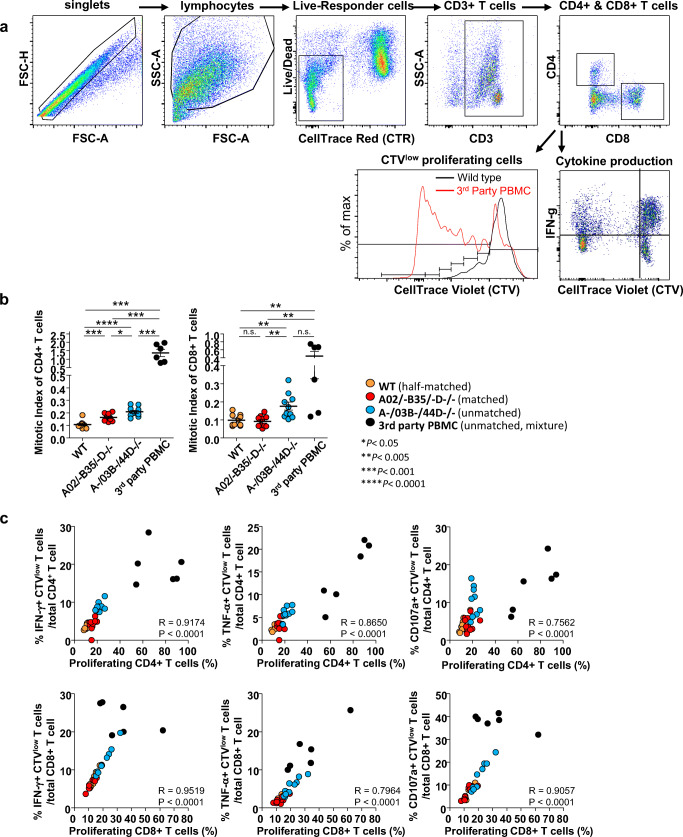
T cell responses to genome-edited HLA-A, B hemizygous cells in vitro*.* (**a**) The gating strategy for analysis of the mixed lymphocyte reaction is shown. Gates were applied to exclude dead cells and target cells, to obtain CD3^+^ T cells and subsequently CD4^+^ or CD8^+^ T cells. Detailed analysis for CTV^low^ proliferating cells in response to antigen is shown by histogram. The mitotic index was calculated as the number of mitotic events/number of absolute precursors. For each type of T cell, the mitotic index and cytokine production were determined by flow cytometric analysis. (**b**) Mitotic indices of proliferating CD8^+^ and CD4^+^ T cells in the presence of different cell types are shown. (**c**) Correlation between cytokine production and proliferation in CD4^+^ and CD8^+^ T cells. *p < 0.05, **p < 0.005, ***p < 0.001, ****p < 0.0001 followed by Spearman’s nonparametric test

T cell proliferative activity was determined by flow cytometry based on a gating strategy (Fig. 4a). The percentage of each cell type was converted to a mitotic index [39] to accurately calculate the reaction. The mitotic index is a measure of cellular proliferation and is extrapolated from mitotic events by dividing the number of precursors by the total number of cells. A mixture of ten unmatched PBMC populations (third party PBMCs) was used as a positive control. The proliferation of allo-reactive T cells, especially CD8^+^ cytotoxic T cells, was significantly increased against HLA class I unmatched (A−/03B−/44D−/−) target cells compared to that against HLA class I matched (A02/−B35/−D−/−) or WT (half-matched) target cells (Fig. 4b).

Furthermore, like the T cell proliferation results, production of cytokines (e.g., IFN-γ and TNF-α) and degranulation of T cells (measured by the CD107a surface antigen level) were higher when the responder T cells were incubated with HLA class I unmatched (A02/−B35/−D−/−) or third party PBMCs than when HLA class I matched target cells (A02/−B35/−D−/−) or WT cells were used (Fig. 4c). Convincingly, our MLR results for both T cell subsets displayed a high correlation between T cell proliferation and cytokine secretion and degranulation of T cells (Fig. 4c). Together, the results of this study showed that HLA-edited matched cells elicited significantly reduced T cell responses compared to HLA-unmatched cells.

### Estimation of Population Coverage

**Fig. 5:**
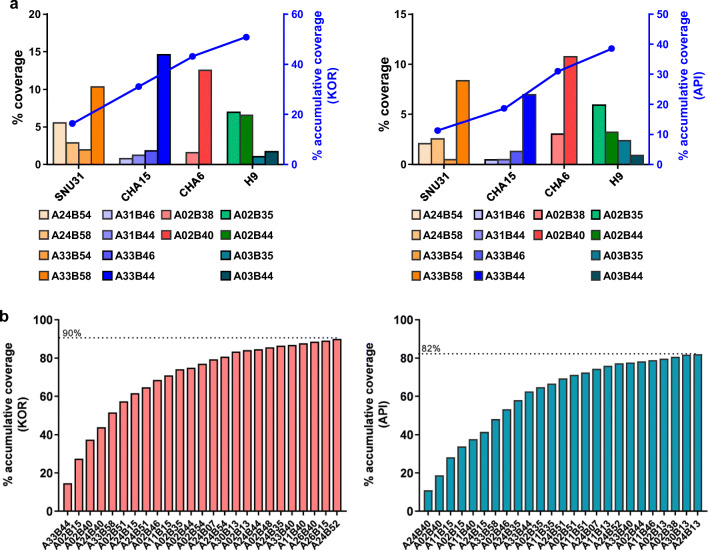
Estimation of population coverage. (**a**) The proportions of Korean (KOR) and Asian-Pacific Islander (API) populations matched to our small HLA hemizygous hESC library are shown. Our HLA-A, B hemizygous hESC library, made from four established hESC lines, covers >50% of the KOR population and about 40% of the API population. (**b**) Twenty five different hemizygous hESC lines from 16 pre-established hESCs can cover ~90% of the KOR population. Likewise, 25 different hemizygous hESC lines from a different set of 13 pre-established hESCs can cover ~82% of the API population

We calculated how many people in two populations would be covered by the 14 HLA-edited hESC mini libraries derived from the four hESC lines (H9, CHA15, CHA6, and SNU31). We found that the 14 HLA-AB hemizygous and DRB1 biallelic knockout hESC lines could cover more than 50% of the Korean population and about 38% of the Asian-Pacific population [29] (Fig. 5a). Two of our 14 HLA-edited hemizygous hESC lines had HLA haplotypes A*33/B*44 and A*33/B*58, which are among the five most common HLA haplotypes in the Korean population [40].

Currently, many well characterized hESC lines are available from Korea and China [28, 41]. Based on the HLA types of a total of 202 hESC lines described in the two reports, we estimated that HLA-editing of 16 pre-established hESC lines would be needed to cover >90% of the Korean population and 13 pre-established hESC lines would be needed to cover >82% of the Asian population: 25 HLA-AB haplotype combinations derived from the 16 and 13 heterozygous hESC lines would be enough to cover >90% of Korean and >82% of Asian populations, respectively (Fig. 5b, Table S2 and S3). In addition, 19 pre-established hESC lines are needed to cover >90% of the API population (Table S2).

Our estimation indicates that only a small number of established hESC lines would be sufficient to generate an HLA-AB haplotype library matching most Asian people, let alone the Korean population.

## Discussion

Successful and safe allogeneic transplantation requires a sophisticated balance between evasion and recognition of the engrafted donor cells by host immune surveillance, and this balance varies depending on the circumstances. Immune evasion is required for successful engraftment and prolonged integration of the transplanted donor cells into the host environment. On the other hand, immune recognition is also warranted in certain cases, such as malignancy or opportunistic infection of the engrafted cells. In that sense, extensive editing of many immune-related molecules including HLAs must be carefully evaluated for safety before broader use of the cells in the clinic.

One approach for avoiding immune rejection of an allograft is to use HLA-matched donor cells for transplantation. In contrast to organ transplantation, for which there is a shortage of human donor organs, banking of a wide variety of hESC lines with homozygous HLA types would provide an unlimited “off-the-shelf” cell source for immune-compatible transplantation.

The number of HLA homozygous cell lines required to cover a large population of people depends on the number of HLA genes that must be matched as well as the number of HLA gene subtypes used for donor-recipient matching: For example, fewer cell lines are needed to match only two HLA genes (i.e., *HLA-A* and *-B*) than to match three HLA genes (i.e., *HLA-A, -B,* and *-C*). In our study, we used the matching strategy of conventional organ transplantation (i.e., matching HLA-A, -B and -DRB1). To this end, we generated matching donor cells by monoallelic knockout of the *HLA-A* and *-B* genes (hemizygous state) and biallelic knockout of the *HLA-DRB1* gene (null state). In this way, four hESC lines with different combinations of hemizygous (homozygous-like) *HLA-A* and *-B* genes can be generated from an hESC line heterozygous for HLA, which enabled us to establish an “off-the-shelf” hESC library that covers a large number of people using already established hESCs without further embryo destruction.

Among pluripotent stem cells, we focused on hESCs that were obtained from blastocysts formed during early embryogenesis, thus avoiding potential reprogramming-induced genetic and epigenetic aberrations often observed in iPSCs [7–12, 42].

Currently, a large number of hESC lines have been established worldwide. A total of 414 hESC lines have been registered in the NIH Human Embryonic Stem Cell Registry (https://grants.nih.gov/stem_cells/registry/current.htm), 764 lines in the Human Pluripotent Stem Cell Registry (hPSCreg) (https://hpscreg.eu/news/single_news?id=104), 23 lines in the UK Stem Cell Bank (https://www.nibsc.org/science_and_research/advanced_therapies/uk_stem_ cell_bank/cell_line_catalogue/research_grade_stem_cell_lines.aspx), and 94 lines in the Korea Stem Cell Distribution & Education System (http://www.stemcellkorea.org/2_stemcell_page_ 02.php). Furthermore, the number of clinical grade hESC lines has also been increasing, with over 20 clinical grade hESC lines registered in the UK Stem Cell Bank alone (https://www.nibsc.org/science_and_research/ advanced_therapies/uk_stem cell_bank/cell_line_catalogue/eutcd_grade_.aspx) [43–46]. Therefore, it would be feasible to establish a sufficient number of HLA-A, B hemizygous hESC lines to match a large fraction of the human population from these pre-established hESCs. Although generation and characterization of dozens of cGMP-grade hemizygous hESC lines is not trivial task, our study provides a useful platform to establish “off-the-shelf” immune-compatible cell bank.

Recent efforts to achieve immune-compatible hPSC lines included the removal of all HLA class I and II gene function by knocking out the *B2M* and *CIIA* genes, respectively [17–19]. To avoid NK cell-mediated killing, HLA-E gene was introduced (B2M KO/HLA-E) [18]. In addition, some studies introduced ‘don’t-eat-me’ genes such as *PD-L1* and *CD47* so that host immune surveillance could be evaded [17, 19]. However, evasion of host immune surveillance is potentially dangerous, especially if the engrafted cells later become malignant or infected with a pathogen. Xu et al. generated HLA-edited iPSCs in which the genes encoding HLA-A and -B, two major targets of cytotoxic T cells, were removed biallelically, whereas one copy of HLA-C was deleted monoallelically. The resulting HLA-edited iPSCs (HLA-A (−/−), -B (−/−), and -C (+/−)) were not recognized by the host’s T cell- and NK cell-mediated immune surveillance. The authors also generated HLA-AB-hemizygous iPSCs (but no null DRB1), which caused no significant T cell response. However, the response of NK cells to these iPSCs was not examined.

Although both iPSCs and hESCs are pluripotent stem cells, it cannot be assumed that hESCs will generate the same immune responses as iPSCs. Because iPSCs display varying gene expression profiles (potentially including differential expression of minor histocompatibility genes, among other relevant genes) as a result of the inconsistent nature of the reprogramming process, as well as reprogramming-induced genomic aberrations, iPSCs will potentially cause different immune responses than hESCs.

In our study, we demonstrated that our HLA-edited hESCs (hemizygous HLA-A, -B and null HLA-DRB1) elicited low T and NK cell-mediated immune responses, suggesting potentially low levels of immune rejection after engraftment. Because our HLA-edited hESCs still retain all classical HLA class I antigens including HLA-A and -B, these cells and their derivatives would be efficiently recognized for destruction by the host immune system in cases of pathogenic infection or oncogenic transformation. Therefore, our strategy would provide safe donor cells that allow the immune compatibility required for efficient cell engraftment, but still retain the potential for host immune surveillance. At this stage, we have not compared our hemizygous lines with B2M KO/HLA-E cell line [18] in terms of immune evasion and safety. This issue needs to be addressed in a future study.

Together, the results of this study suggest that our HLA-editing strategy could expedite the translation of cell replacement interventions from bench to bedside by providing “off-the-shelf” immune-compatible pluripotent stem cells. Because of their lack of reprogramming-induced genomic and epigenetic abnormalities, hESCs represent a gold standard among pluripotent stem cells, making HLA-edited hESCs especially promising therapeutically.

## Supplementary Information

Supplemental Fig. 1 HLA haplotype information for the HLA-edited cell lines and HLA gene-specific sgRNAs. Related to Fig. 1**.** (**a**) HLA haplotype information for CHA15, CHA6, H9, and SNU31 hESCs. CHA15 and CHA6 haplotype information is from [28]. H9 haplotype information is from [27]. (**b**) A list of sgRNA sequences targeting the *HLA-DRB1*, *HLA-A*, and *HLA-B* genes in the CHA15, CHA6, H9, and SNU31 hESC lines. (PDF 441 kb)

Supplemental Fig. 2 Indel patterns in the HLA-edited hESC clones generated in this study. Related to Fig. 1**.** Detailed indel patterns from genome-edited (**a**) CHA15, (**b**) SNU31, (**c**) CHA6, and (**d**) H9_B2M−/−DRB1−/− hESCs are shown. Deleted nucleotides are shown as dashes and inserted nucleotides are denoted as bold lower-case letters. (PDF 153 kb)

Supplemental Fig. 3 Whole genome sequencing of H9_A02/−B35/−D−/− and H9_WT hESC clones. Related to Fig. 2**.** Genomic DNAs from the H9_A02/−B35/−D−/− clone and H9_WT hESCs were sequenced by Illumina Hiseq X10. First, total single nucleotide variants (SNVs) and small indels were identified by Isaac Variant Caller, after which we filtered out annotated variants based on the SNP database (dbSNP) and SNPs existing in the H9_WT genomic DNA. We found 13,423 SNPs in the H9_A02/−B35/−D−/− clone. We compared these SNPs to the potential off-target sites found by Cas-OFFinder by allowing up to 7 mismatches or up to 5 mismatches with DNA or RNA bulges. None of the variants were found at the estimated (potential off-target) locations. (PDF 47 kb)

Supplemental Fig. 4 Characterization of endothelial cells (ECs) derived from HLA-edited hESCs. Related to Fig. 2**.** ECs differentiated from both WT and HLA-edited (A02/−B35/−D−/− and A−/03B−/44D−/−) H9 hESCs were immunostained for the expression of representative EC markers, CD31 and VWF (Von Willebrand factor). Scale bars: 20 μm. (PDF 156 kb)

Supplemental Fig. 5 Expansion and activation of NK cells for use in an NK cytotoxicity assay. Related to Fig. 3**.** (**a**) Flow cytometry data showed that the frequency of CD3^−^CD56^+^ NK cells was elevated after activation and expansion of NK cells. The proportion of expanded CD3^−^CD56^+^ NK cells reached a plateau of approximately 73% of the total cell population after cultivation. (**b**) Several markers for activated NK cells, including CD16, NKG2D, CD94, and CD69, were upregulated on the expanded NK cells. All parameters were gated on CD3^−^CD56^+^ NK cells. Expression levels before and after cultivation are shown in gray and blue, respectively. ***P<0.001. (PDF 203 kb)

Supplemental Fig. 6 Cytokine production by NK cells in response to co-culture with various types of target cells including HLA-edited ECs. Related to Fig. 3. (**a**) Representative flow cytometry data measuring IFN-γ and TNF-α expression from responder NK cells after co-culturing with various target cells are shown. (**b**) IFN-γ and/or TNF-α expression by NK cells was measured by flow cytometry at an E:T ratio of 1:1. Results are shown as means ± SEMs. (PDF 258 kb)

Supplemental Fig. 7 Characterization of HLA-edited target cells (ECs) used for T cell proliferation and cytokine producing assays. Related to Fig. 4. (**a**) HLA-type information for the target cells (HLA-edited ECs and 3rd party pooled PBMCs) and responder PBMCs. The 3rd party pooled PBMCs, a mixture of PBMCs derived from 10 healthy people, were used as positive control target cells. (**b**) The percentage of target ECs expressing CD31 was assessed by flow cytometry. Data are represented as means ± SEMs (*n* = 5). ***p< 0.001, one-way ANOVA, followed by Tukey’s test. (**c**) The percentage of target ECs expressing HLA-ABC was assessed by flow cytometry. Data are represented as means ± SEMs (n = 5). ***p < 0.001, one-way ANOVA, followed by Tukey’s test. (**d**) Phenotypes of expanded T cells derived from PBMCs from three healthy donors. Total CD3^+^CD56^−^ T cells were delineated to naïve and memory phenotypes, including CCR7 and CD45RA expression. Total CD3^+^CD56^−^ T cells were divided into CD4^+^ T cells and CD8^+^ T cells, gated on live CD45^+^ lymphocytes. (PDF 614 kb)

Table S1 (XLSX 13 kb)

Table S2 (XLSX 25 kb)

Table S3 (XLSX 11 kb)
