# Supplementary material for: Off-the-Shelf, Immune-Compatible Human Embryonic Stem Cells Generated Via CRISPR-Mediated Genome Editing
Source: Stem Cell Rev Rep. 2021 Jan 9;17(3):1053–67. doi: 10.1007/s12015-020-10113-7 (PMC8166669; doi:10.1007/s12015-020-10113-7)
Supplement: Supplementary file 1 — HLA haplotype information for the HLA-edited cell lines and HLA gene-specific sgRNAs. Related to Fig. 1. (a) HLA haplotype information for CHA15, CHA6, H9, and SNU31 hESCs. CHA15 and CHA6 haplotype information is from [28]. H9 haplotype information is from [27]. (b) A list of sgRNA sequences targeting the HLA-DRB1, HLA-A, and HLA-B genes in the CHA15, CHA6, H9, and SNU31 hESC lines. (PDF 441 kb) [file 12015_2020_10113_MOESM1_ESM.pdf]

Supplemental Fig. 1

a

Target cell	Original haplotype		
	HLA-A	HLA-B	HLA-DRB1
H9	A*02<br>A*03	B*35<br>B*44	DRB1*15<br>DRB1*16
CHA15	A*31<br>A*33	B*44<br>B*46	DRB1*04<br>DRB1*13
SNU31	A*24<br>A*33	B*54<br>B*58	DRB1*04<br>DRB1*13
CHA6	A*02<br>A*02	B*38<br>B*40	DRB1*09<br>DRB1*11

HLA-A,-B monoallelic knockout

HLA-DRB1 biallelic knockout

HLA-engineered hESC lines	Final haplotype		Subypes of HLA-A, -B, DRB1
	HLA-A	HLA-B	
H9-hemi-line 1	A*02 ~~A*03~~	B*35 ~~B*44~~	A02/-B35/-D-/-
H9-hemi-line 2	~~A*02~~ A*03	~~B*35~~ B*44	A-/03B-/44D-/-
H9-hemi-line 3	A*02 ~~A*03~~	~~B*35~~ B*44	A02/-B-/44D-/-
H9-hemi-line 4	~~A*02~~ A*03	B*35 ~~B*44~~	A-/03B35/-D-/-
CHA15-hemi-line 1	A*31 ~~A*33~~	B*44 ~~B*46~~	A31/-B44/-D-/-
CHA15-hemi-line 2	~~A*31~~ A*33	~~B*44~~ B*46	A-/33B-/46D-/-
CHA15-hemi-line 3	A*31 ~~A*33~~	~~B*44~~ B*46	A31/-B-/46D-/-
CHA15-hemi-line 4	~~A*31~~ A*33	B*44 ~~B*46~~	A-/33B44/-D-/-
SNU31-hemi-line 1	A*24 ~~A*33~~	B*54 ~~B*58~~	A24/-B54/-D-/-
SNU31-hemi-line 2	~~A*24~~ A*33	~~B*54~~ B*58	A-/33B-/58D-/-
SNU31-hemi-line 3	A*24 ~~A*33~~	~~B*54~~ ~~B*58~~	A24/-B-/58D-/-
SNU31-hemi-line 4	~~A*24~~ A*33	B*54 B*58	A-/33B54/-D-/-
CHA6-hemi-line 1	A*02 A*02	B*38 ~~B*40~~	A02/02B38/-D-/-
CHA6-hemi-line 2	A*02 A*02	~~B*38~~ B*40	A02/02B-/40D-/-

b

Cell name	HLA-A		HLA-B		HLA-DRB1	
	Haplotype	Target sequence (5’ -> 3’)	Haplotype	Target sequence (5’ -> 3’)	Haplotype	Target sequence (5’ -> 3’)
H9	A*02	ACCCTCGTCCTGCTACTCTCGGG	B*35	CGTCCTCCTGCTGCTCTGGGGGG	DRB1*15	CCTGAACGGCCAGGAAGAGAAGG
	A*03	ACCCTCCTCCTGCTACTCTCGGG	B*44	CCTCCTCCTGCTGCTCTGGGGGG	DRB1*16	
CHA15	A*31	ACAGCGACGCCGCGAGCCAGAGG	B*44	AGTTGTGGTCATCGGAGCTGTGG	DRB1*04	CACTGTCAGAGCTGCCATGCAGG
	A*33		B*46		DRB1*13	CACTGTCAGAACTGCCATGCAGG
SNU31	A*24	ACCCTCGTCCTGCTACTCTCGGG	B*54	CCTCCTCCTGCTGCTCTGGGGGG	DRB1*04	TCTTCTCTTCCTGGCCGTTCCGG
	A*33	ACCCTCCTCCTGCTACTCTTGGG	B*58	CGTCCTCCTGCTGCTCTGGGGGG	DRB1*13	TCTTCTCTTCCTGGCCATTCCGG
CHA6	A*02	-	B*38	AGTTGTGGTCATCGGAGCTGTGG	DRB1*09	TGATGCTGGAAACAGTTCCTCGG
	A*02		B*40		DRB1*11	
