# Supplementary material for: Off-the-Shelf, Immune-Compatible Human Embryonic Stem Cells Generated Via CRISPR-Mediated Genome Editing
Source: Stem Cell Rev Rep. 2021 Jan 9;17(3):1053–67. doi: 10.1007/s12015-020-10113-7 (PMC8166669; doi:10.1007/s12015-020-10113-7)
Supplement: Supplementary file 2 — Indel patterns in the HLA-edited hESC clones generated in this study. Related to Fig. 1. Detailed indel patterns from genome-edited (a) CHA15, (b) SNU31, (c) CHA6, and (d) H9_B2M−/−DRB1−/− hESCs are shown. Deleted nucleotides are shown as dashes and inserted nucleotides are denoted as bold lower-case letters. (PDF 153 kb) [file 12015_2020_10113_MOESM2_ESM.pdf]

Supplemental Fig. 2

a

CHA15

```
WT               A*31  CGACAGCGACGCCGCGAGCCAGAGGATGGAGCCGC
A31/33B44/46     A*33  CGACAGCGACGCCGCGAGCCAGAGGATGGAGCCGC
D+/+             B*44  GCAGTTGTGGTCATCGGAGCTGTGGTCGCTGCTGT
                 B*46  GCAGTTGTGGTCATCGGAGCTGTGGTCGCTACTGT
                 DRB1  GGTGATGCTGGAAACAGTTCCTCGGAGTGGAGAGG

A-/33B-/46D-/-   A*31  CGACAGCGAC-----------------GGAGCCGC    -17
                 B*44  GCAGTTGTGGTCATCGGAGcCTGTGGTCGCTACTGT   +1
                 DRB1  GGTGATGCTGGAAACAG-TCCTCGGAGTGGAGAGG    -1
                 DRB1  GGTGATGCT--------------GGAGTGGAGAGG    -14

A31/-B44/-D-/-   A*33  CGAC-------------------------AGCCGC    -25
                 B*46  GCAGTTGTGGTCATCGGAG-TGTGGTCGCTACTGT    -1
                 DRB1  GGTGATGCTGGAAACAG-TCCTCGGAGTGGAGAGG    -1
                 DRB1  GGTGATGCT--------------GGAGTGGAGAGG    -14

A31/-B44/-D-/-   A*33  CGACA-----------AGCCAGAGGATGGAGCCGC    -11
                 B*44  GCAGTTGTGGTCATCGGAGcCTGTGGTCGCTACTGT   +1
                 DRB1  GGTGATGCTGGAAACAG-TCCTCGGAGTGGAGAGG    -1
                 DRB1  GGTGATGCT--------------GGAGTGGAGAGG    -14

A-/33B44/-D-/-   A*31  CGACAGCGA-----CGAACCAGAGGATGGAGCCGC    -5
                 B*46  GCAGTTGTGGTCATCGGAG-TGTGGTCGCTACTGT    -1
                 DRB1  GGTGATGCTGGAAACAG-TCCTCGGAGTGGAGAGG    -1
                 DRB1  GGTGATGCT--------------GGAGTGGAGAGG    -14
```

b

SNU31

```
WT               A*24  GAACCCTCGTCCTGCTACTCTCGGGGGCCCTGGCC
A24/33B54/58     A*33  GAACCCTCCTCCTGCTACTCTTGGGGGCCCTGGCC
D+/+             B*54  ACCCTCCTCCTGCTGCTCTGGGGGGCCCTGGCCCT
                 B*58  ACCGTCCTCCTGCTGCTCTGGGGGGCAGTGGCCCT
                 D*04  TTCCGGAACGGCCAGGAAGAGAAGACTGGGGTGGT
                 D*13  TTCCGGAATGGCCAGGAAGAGAAGACTGGGGTGGT

A-/33B-/58D-/-   A*24  GAACCCTCGTCCTGCTA--CTCGGGGGCCCTGGCC    -2
                 B*54  ACCCTCCTCCTGCTGCTCTtGGGGGGCCCTGGCCCT   +1
                 D*04  TTCCGGAAgagaaccaggCGGCCAGGAAGAGAAGA    +10
                 D*13  TTCCGGAA----------GAGAAGACTGGGGTGGT    -10

A-/33B54/-D-/-   A*24  GAACCCTCGTCCTGCTAC----------------C    -16
                 B*58  ACCGTCCTCCTGCTGCTCTtGGGGGGCAGTGGCCCT   +1
                 D*04  TTCCGGAAgagaaccaggCGGCCAGGAAGAGAAGA    +10
                 D*13  TTCCGGAA----------GAGAAGACTGGGGTGGT    -10

A24/-B54/-D-/-   A*33  GAACCCTCCTCCTGCTACTtCTTGGGGGCCCTGGCC   +1
                 B*58  ACCGTCCTCCTGCTGC--AGGGGGGCAGTGGCCCT    -2
                 D*04  TTCCGGAAgagaaccaggCGGCCAGGAAGAGAAGA    +10
                 D*13  TTCCGGAA----------GAGAAGACTGGGGTGGT    -10

A24/-B-/58D-/-   A*33  GAACCCTCCTCCTGCTACTtCTTGGGGGCCCTGGCC   +1
                 B*54  ACCCTCCTCCTGCTGCTCTtGGGGGGCCCTGGCCCT   +1
                 D*04  TTCCGGAAgagaaccaggCGGCCAGGAAGAGAAGA    +10
                 D*13  TTCCGGAA----------GAGAAGACTGGGGTGGT    -10
```

c

CHA6

```
WT               B*38  AGTTGTGGTCATCGGAGCTGTGGTCGCTGCTGTGATGTGTAGGAGGAA
A02/02B38/40/    B*40  AGTTGTGGTCATCGGAGCTGTGGTCGCTGCTGTGATGTGTAGGAGGAA
D+/+             DRB1  TGATGCTGGAAACAGTTCCTCGGAGTGGAGAAGTTTACACCTGCCAAG
                 DRB1  TGATGCTGGAAACAGTTCCTCGGAGTGGAGAGGTTTACACCTGCCAAG

A02/02B-/40D-/-  B*38  AGTTGTGGTCATCGGA-CTGTGGTCGCTGCTGTGATGTGTAGGAGGAA    -1
                 DRB1  TGATGCTGGAAACAGTT-CTCGGAGTGGAGAGGTTTACACCTGCCAAG    -1
                 DRB1  TGATGC-----------------------------------------G    -41

A02/02B38/-D-/-  B*40  AGTTGTGGTCATCG--aCTGTGGTCGCTGCTGTGATGTGTAGGAGGAA    -2
                 DRB1  TGATGCTGGAAACAGTT-CTCGGAGTGGAGAGGTTTACACCTGCCAAG    -1
                 DRB1  TGATGC-----------------------------------------G    -41
```

d

H9_B2M Knockout

```
WT               B2M   GGCCTTAGCTGTGCTCGCGCTACTCTCTCTTTCTG
B2M+/+D-/-       DRB1  TTCCTGAACGGCCAGGAAGAGAAGGCTGGGATGGT

B2M-/-D-/-       B2M   GGCCTTAGaCTGTGCTCGCGCTACTCTCTCTTTCTG   +1
                 DRB1  TTCCTGAACGGCCAGGAA-----GGCTGGGATGGT    -5
                 DRB1  TTCCTGAACGGCCA-----AGAAGGCTGGGATGGT    -5
```
