# Supplementary material for: Off-the-Shelf, Immune-Compatible Human Embryonic Stem Cells Generated Via CRISPR-Mediated Genome Editing
Source: Stem Cell Rev Rep. 2021 Jan 9;17(3):1053–67. doi: 10.1007/s12015-020-10113-7 (PMC8166669; doi:10.1007/s12015-020-10113-7)
Supplement: Supplementary file 3 — Whole genome sequencing of H9_A02/−B35/−D−/− and H9_WT hESC clones. Related to Fig. 2. Genomic DNAs from the H9_A02/−B35/−D−/− clone and H9_WT hESCs were sequenced by Illumina Hiseq X10. First, total single nucleotide variants (SNVs) and small indels were identified by Isaac Variant Caller, after which we filtered out annotated variants based on the SNP database (dbSNP) and SNPs existing in the H9_WT genomic DNA. We found 13,423 SNPs in the H9_A02/−B35/−D−/− clone. We compared these SNPs to the potential off-target sites found by Cas-OFFinder by allowing up to 7 mismatches or up to 5 mismatches with DNA or RNA bulges. None of the variants were found at the estimated (potential off-target) locations. (PDF 47 kb) [file 12015_2020_10113_MOESM3_ESM.pdf]

Supplemental Fig. 3

	WT	A02/-B35/-D-/- #51
(1) Total No. of SNVs	4322323	4266359
(2) Unique SNVs after filtering out overlapped by WT		111149
(3) Unique SNVs excluding known SNVs in (2)		19616
(4) Unique Indels excluding SNPs in (3)		13423
Overlapped indels in (4) with potential off-target positions		
Cas-Offinder candidates of		**A03**
maximum 7nt mismatches with NRG PAM (662829 sites)		0
maximum 5nt mismatches and up to 2nt DNA bulges with NRG PAM (686920 sites)		0
maximum 5nt mismatches and up to 2nt RNA bulges with NRG PAM (2722426 sites)		0
Cas-Offinder candidates of		**B44**
maximum 7nt mismatches with NRG PAM (673761 sites)		0
maximum 5nt mismatches and up to 2nt DNA bulges with NRG PAM (806688 sites)		0
maximum 5nt mismatches and up to 2nt RNA bulges with NRG PAM (2936919 sites)		0
Cas-Offinder candidates of		**DRB1**
maximum 7nt mismatches with NRG PAM (520860 sites)		0
maximum 5nt mismatches and up to 2nt DNA bulges with NRG PAM (517998 sites)		0
maximum 5nt mismatches and up to 2nt RNA bulges with NRG PAM (3306836 sites)		0
