# Supplementary figures and images for: Off-the-Shelf, Immune-Compatible Human Embryonic Stem Cells Generated Via CRISPR-Mediated Genome Editing
Source: Stem Cell Rev Rep. 2021 Jan 9;17(3):1053–67. doi: 10.1007/s12015-020-10113-7 (PMC8166669; doi:10.1007/s12015-020-10113-7)

Supplemental Fig. 4

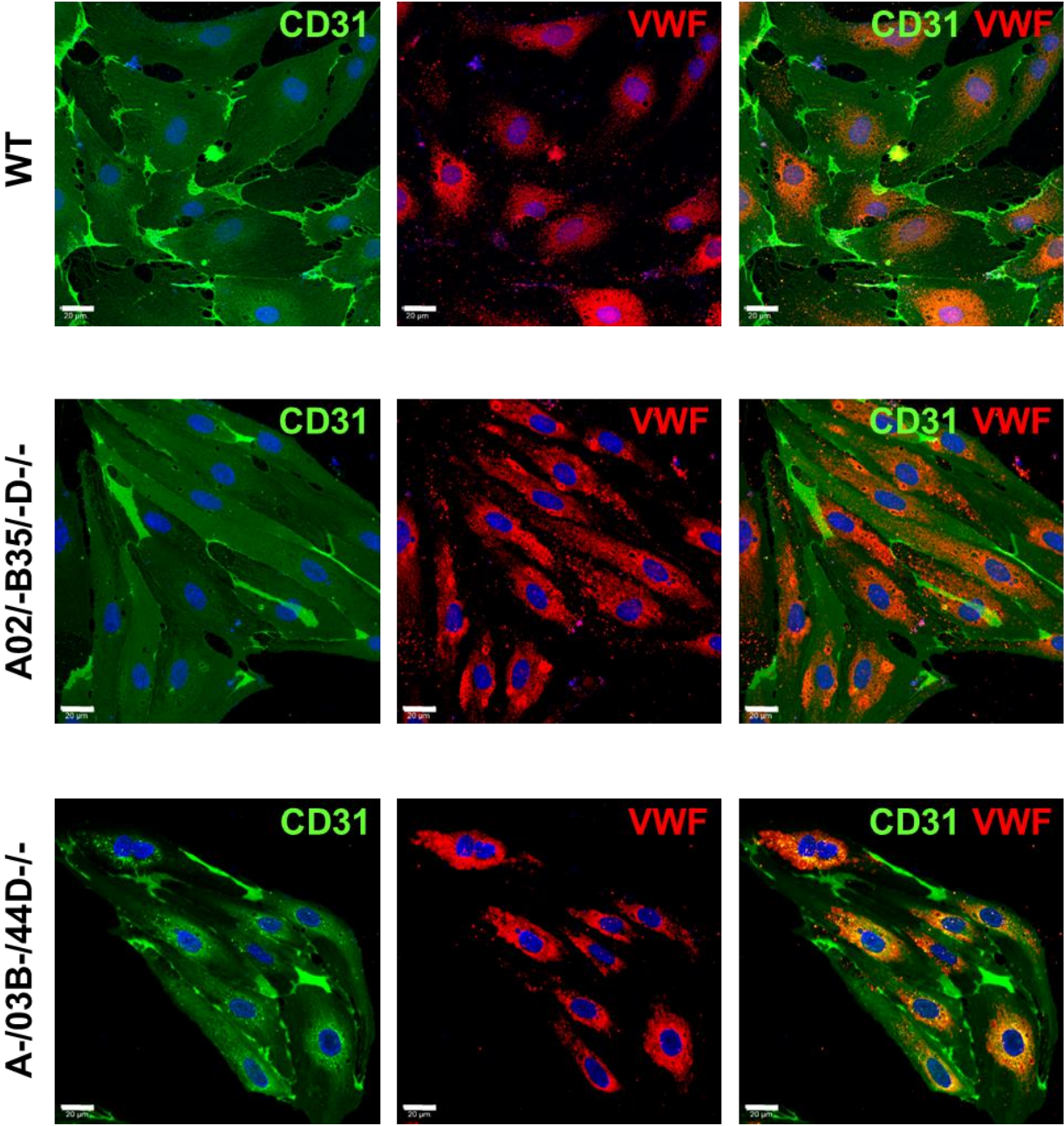

Supplement: Supplementary file 4 — Characterization of endothelial cells (ECs) derived from HLA-edited hESCs. Related to Fig. 2. ECs differentiated from both WT and HLA-edited (A02/−B35/−D−/− and A−/03B−/44D−/−) H9 hESCs were immunostained for the expression of representative EC markers, CD31 and VWF (Von Willebrand factor). Scale bars: 20 μm. (PDF 156 kb) [file 12015_2020_10113_MOESM4_ESM.pdf]

Supplemental Fig. 5

a

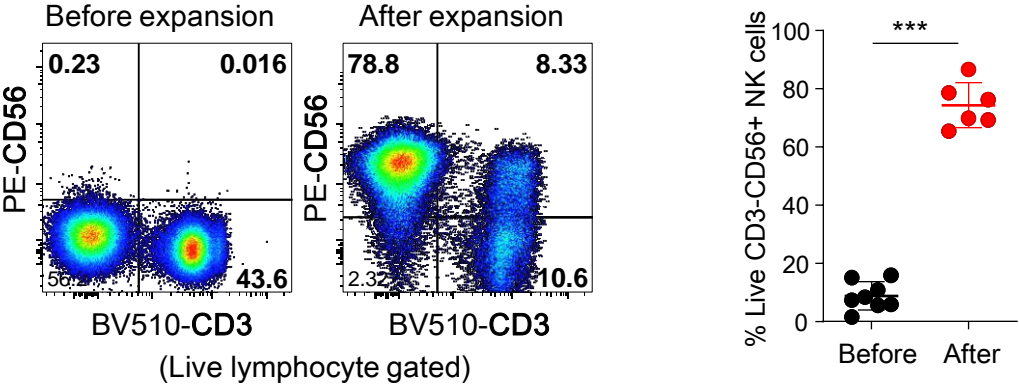

b

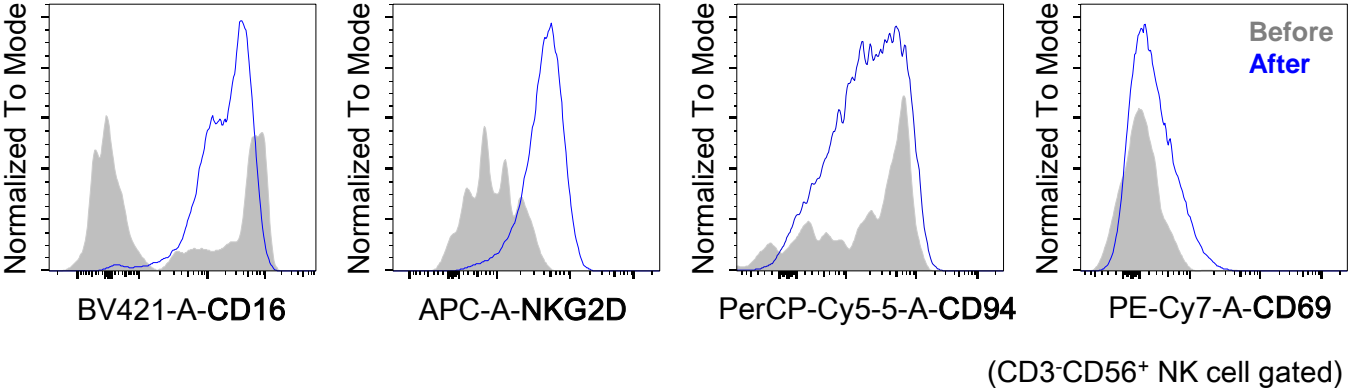

Supplement: Supplementary file 5 — Expansion and activation of NK cells for use in an NK cytotoxicity assay. Related to Fig. 3. (a) Flow cytometry data showed that the frequency of CD3−CD56+ NK cells was elevated after activation and expansion of NK cells. The proportion of expanded CD3−CD56+ NK cells reached a plateau of approximately 73% of the total cell population after cultivation. (b) Several markers for activated NK cells, including CD16, NKG2D, CD94, and CD69, were upregulated on the expanded NK cells. All parameters were gated on CD3−CD56+ NK cells. Expression levels before and after cultivation are shown in gray and blue, respectively. ***P<0.001. (PDF 203 kb) [file 12015_2020_10113_MOESM5_ESM.pdf]

Supplemental Fig. 6

a

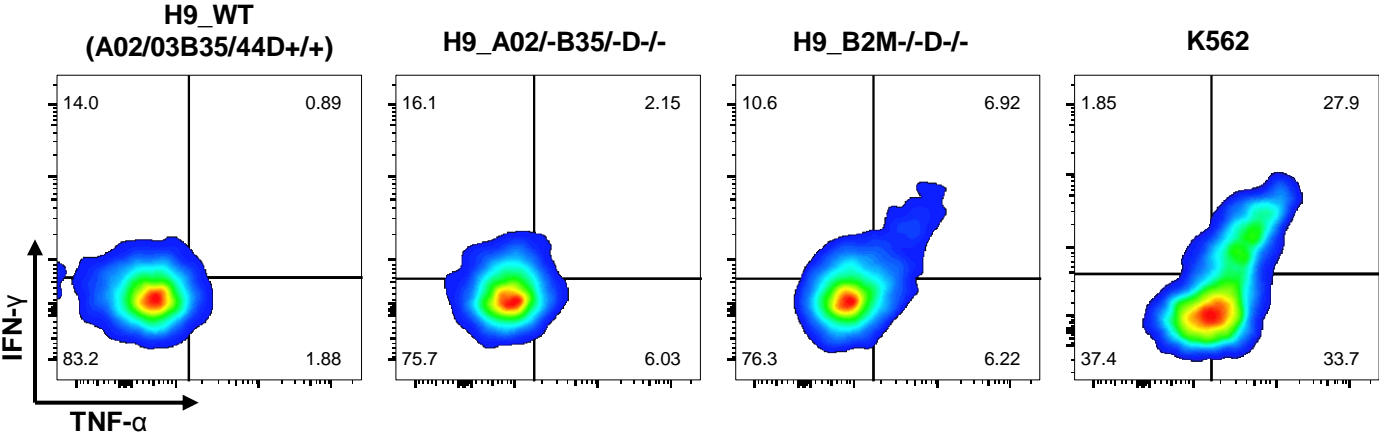

b

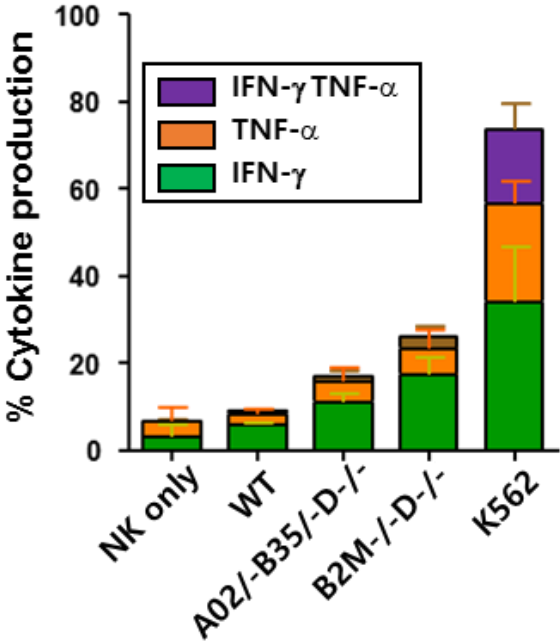

Supplement: Supplementary file 6 — Cytokine production by NK cells in response to co-culture with various types of target cells including HLA-edited ECs. Related to Fig. 3. (a) Representative flow cytometry data measuring IFN-γ and TNF-α expression from responder NK cells after co-culturing with various target cells are shown. (b) IFN-γ and/or TNF-α expression by NK cells was measured by flow cytometry at an E:T ratio of 1:1. Results are shown as means ± SEMs. (PDF 258 kb) [file 12015_2020_10113_MOESM6_ESM.pdf]

Supplemental Fig. 7

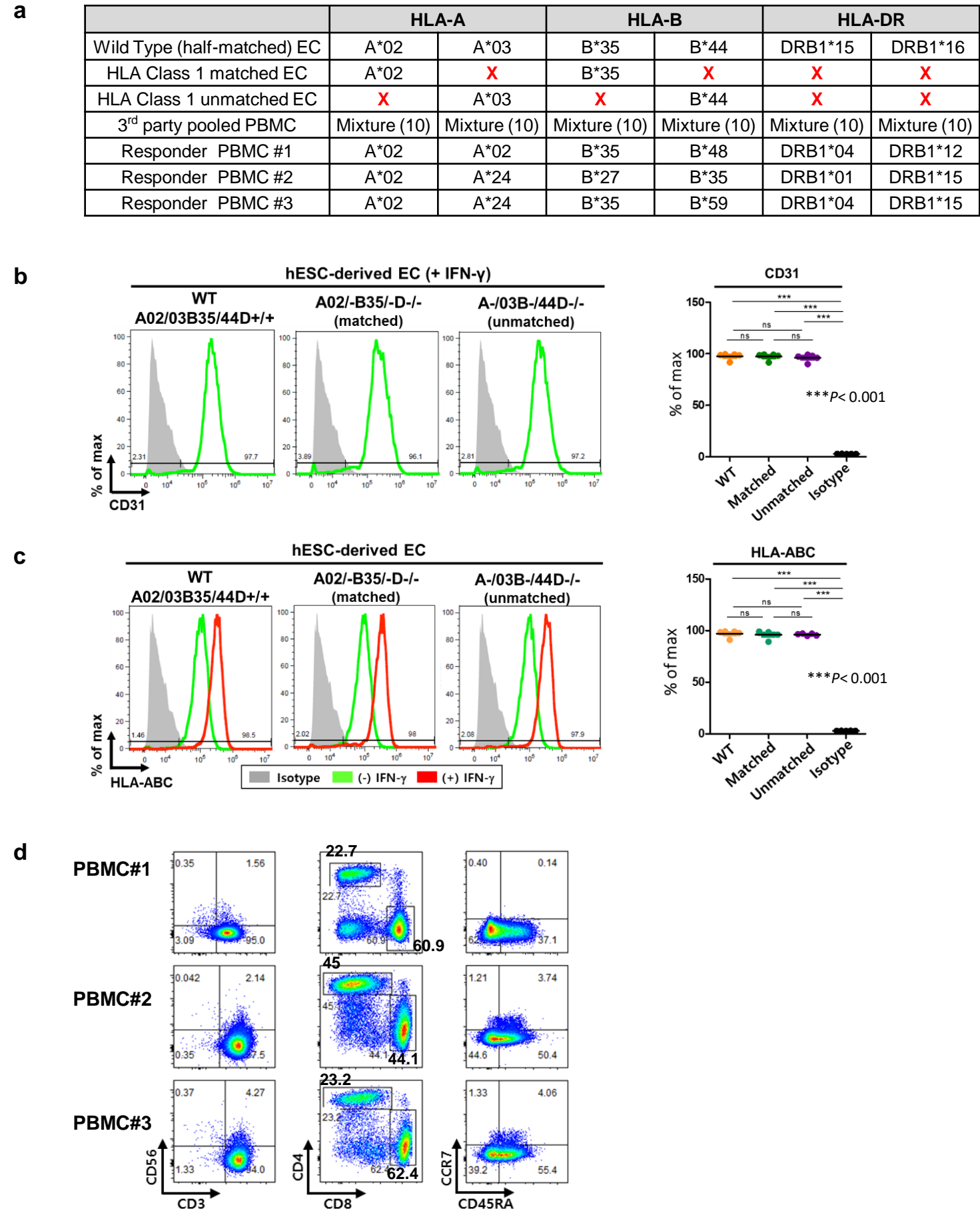

Supplement: Supplementary file 7 — Characterization of HLA-edited target cells (ECs) used for T cell proliferation and cytokine producing assays. Related to Fig. 4. (a) HLA-type information for the target cells (HLA-edited ECs and 3rd party pooled PBMCs) and responder PBMCs. The 3rd party pooled PBMCs, a mixture of PBMCs derived from 10 healthy people, were used as positive control target cells. (b) The percentage of target ECs expressing CD31 was assessed by flow cytometry. Data are represented as means ± SEMs (n = 5). ***p< 0.001, one-way ANOVA, followed by Tukey’s test. (c) The percentage of target ECs expressing HLA-ABC was assessed by flow cytometry. Data are represented as means ± SEMs (n = 5). ***p < 0.001, one-way ANOVA, followed by Tukey’s test. (d) Phenotypes of expanded T cells derived from PBMCs from three healthy donors. Total CD3+CD56− T cells were delineated to naïve and memory phenotypes, including CCR7 and CD45RA expression. Total CD3+CD56− T cells were divided into CD4+ T cells and CD8+ T cells, gated on live CD45+ lymphocytes. (PDF 614 kb) [file 12015_2020_10113_MOESM7_ESM.pdf]
